# A Case of Systemic Lupus Erythematosus Involving the Larynx

**DOI:** 10.7759/cureus.107291

**Published:** 2026-04-18

**Authors:** Arham F Shaikh, Vivek Harkare, Priti R Dhoke, Fahad I Idrees, Asmi R Dhoke

**Affiliations:** 1 Otolaryngology-Head and Neck Surgery, NKP Salve Institute of Medical Sciences and Research Center, Lata Mangeshkar Hospital, Nagpur, IND; 2 Medicine, NKP Salve Institute of Medical Sciences and Research Center, Lata Mangeshkar Hospital, Nagpur, IND; 3 Medicine, Datta Meghe Medical College, Nagpur, IND

**Keywords:** airway compromise, autoimmune disease, laryngeal involvement, odynophagia, systemic lupus erythematosus

## Abstract

Systemic lupus erythematosus (SLE) is a chronic, multisystem autoimmune disorder characterized by the formation of autoantibodies and immune complex deposition, leading to inflammation and damage across various organs. Although the disease commonly involves the skin, joints, kidneys, hematological system, and central nervous system, involvement of the larynx is distinctly rare and often remains undiagnosed. Laryngeal manifestations may vary from mild mucosal edema and dysphonia to severe ulceration, hemorrhage, cricoarytenoid arthritis, and potentially life-threatening airway obstruction. Due to its rarity and nonspecific presentation, laryngeal involvement in SLE may mimic infective, neoplastic, or other inflammatory conditions, posing a diagnostic challenge for otolaryngologists.

We report a case of a 54-year-old female patient who presented with acute onset of odynophagia and a change in voice, which on Hopkins rod evaluation revealed marked supraglottic edema with hemorrhagic bullae and ulcerative lesions. Hematological findings revealed severe anemia and leukopenia, and immunological tests confirmed SLE. She was treated with systemic corticosteroids and supportive therapy, leading to significant clinical and endoscopic improvement. This case highlights the importance of considering autoimmune etiologies such as SLE in patients presenting with unexplained laryngeal edema and ulceration, as early diagnosis and timely management are crucial to prevent airway compromise and associated morbidity.

## Introduction

Systemic lupus erythematosus (SLE) is a chronic autoimmune disease characterized by immune dysregulation and the production of autoantibodies against nuclear and cytoplasmic antigens [1]. It mostly affects women of reproductive age, with a female-to-male ratio of approximately 9-10:1, and demonstrates a higher prevalence in Asian populations, including India [2]. The disease typically follows a relapsing and remitting course with periods of exacerbation and remission [3]. SLE can involve multiple organ systems, most commonly the skin, musculoskeletal system, kidneys, cardiovascular system, nervous system, and hematological system [4]. Ear, nose, and throat (ENT) manifestations are relatively uncommon and often under-recognized in routine clinical practice [5]. Among these, laryngeal involvement is particularly rare, with reported prevalence ranging from 0.3% to 30% depending on diagnostic criteria and inclusion of subclinical cases [6]. Laryngeal manifestations may result from immune complex deposition, vasculitis, mucosal inflammation, or arthritis of the cricoarytenoid joints, presenting as hoarseness, odynophagia, vocal cord palsy, or even life-threatening airway obstruction [7]. The clinical presentation of laryngeal SLE is highly variable and may include hoarseness, odynophagia, dysphagia, cough, stridor, or even acute airway obstruction. Because these symptoms overlap with more common conditions such as acute laryngitis, tuberculosis, fungal infections, and malignancy, diagnosis is often delayed. Awareness of this rare manifestation is therefore essential for early recognition and appropriate management. The present case report aims to contribute to the limited literature on laryngeal involvement in SLE and to emphasize the role of otolaryngologists in identifying this potentially life-threatening condition.

## Case presentation

**Figure 1:**
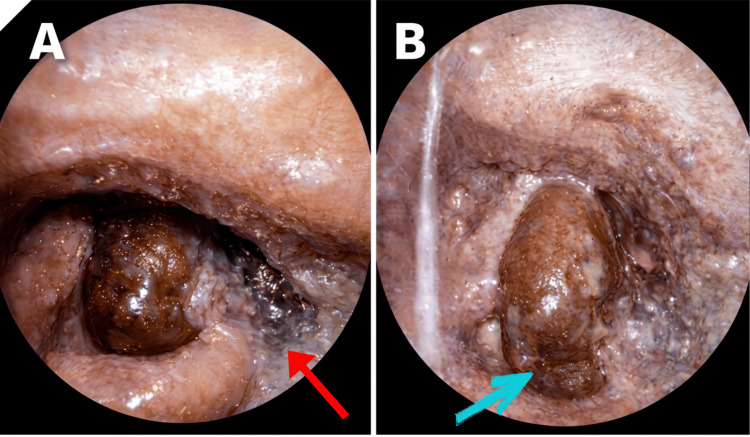
Endoscopic Findings Showing Laryngeal Involvement Endoscopic visualization using the Hopkins rod. (A) The red arrow shows a slough over the lateral pharyngeal wall. (B) The blue arrow shows an edematous epiglottis and right aryepiglottic fold with a hemorrhagic bulla over the right arytenoid.

A 54-year-old female patient presented to the otorhinolaryngology outpatient department with complaints of odynophagia and a change in voice for the past three to four days. The onset of symptoms was acute and progressive. The patient did not present any history of fever, cough, hemoptysis, breathing difficulty, or foreign body ingestion. There was no prior history of trauma to the neck or recent upper respiratory tract infection. The patient did not report any history of tobacco or alcohol use. On general examination, the patient appeared pale and fatigued. Vital signs were stable, and there was no evidence of respiratory distress at presentation. Systemic examination did not reveal any obvious skin rashes, oral ulcers, or joint swelling. Cervical lymphadenopathy was absent. A Hopkins rod endoscopic examination was performed, which revealed gross edema of the epiglottis, right aryepiglottic fold, and right false vocal cord. A hemorrhagic bulla was noted over the right arytenoid. Additionally, an ulcerative lesion covered with slough was observed over the right lateral pharyngeal wall, extending from the free edge of the epiglottis to the right pyriform fossa. The true vocal cords were mobile bilaterally, although visualization was partially obscured by supraglottic edema (Figures 1A-1B).

**Figure 2:**
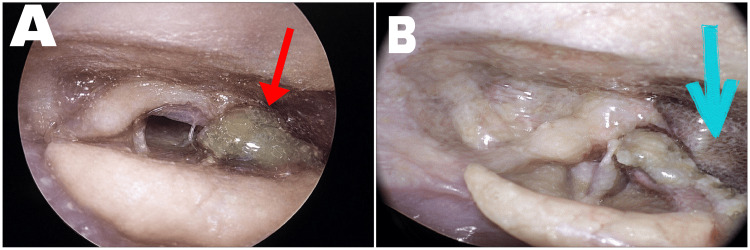
Post-treatment Endoscopic Findings Showing Resolution of Laryngeal Lesions Post-treatment endoscopic images obtained using a Hopkins rod. (A) The red arrow shows a marked reduction in edema and hemorrhagic bulla over the right arytenoid with improvement in surrounding laryngeal inflammation. (B) The blue arrow shows a marked reduction in slough over the lateral pharyngeal wall.

Routine hematological investigations revealed marked leukopenia with a total leukocyte count of (520/µL ) and severe anemia (hemoglobin 4.5 g/dL). Platelet count was within normal limits. Peripheral smear showed normocytic normochromic anemia. Erythrocyte sedimentation rate was elevated. Ultrasonography of the abdomen demonstrated hepatosplenomegaly. In view of the unexplained cytopenias and multisystem involvement, an autoimmune workup was initiated. Antinuclear antibody (ANA) testing by immunofluorescence was strongly positive. Immunological evaluation revealed significant findings supportive of SLE. The extractable nuclear antigen (ENA) panel was positive for anti-Smith (anti-Sm) antibodies, which are highly specific for SLE. ANA by blot test was performed, which furthermore confirmed the diagnosis of SLE. Anti-double-stranded DNA (anti-dsDNA) antibody levels were elevated, further supporting the diagnosis and suggesting disease activity. Complement levels, including C3 and C4, were reduced, indicating active immune complex-mediated disease. Although the patient presented with anemia, there was no laboratory evidence of hemolysis, as serum bilirubin and lactate dehydrogenase (LDH) levels were within the normal range and there was no reticulocytosis; hence, a direct Coombs test was not performed. Antiphospholipid antibody testing (including lupus anticoagulant, anticardiolipin, and anti-β2 glycoprotein I antibodies) was not performed, as there were no clinical features suggestive of antiphospholipid syndrome. Based on the clinical, endoscopic, hematological, and immunological findings, a final diagnosis of laryngeal involvement secondary to SLE was made. The patient was admitted for close monitoring due to the risk of airway compromise. She was started on injectable broad-spectrum antibiotics to cover possible secondary infection and systemic corticosteroids to control autoimmune inflammation. Supportive care included correction of anemia with blood transfusion and adequate hydration. The patient showed marked clinical improvement within a few days of initiation of therapy. Odynophagia and hoarseness gradually reduced. Follow-up endoscopic examination demonstrated a significant reduction in supraglottic edema, resolution of the hemorrhagic bulla, and healing of the ulcerative lesion with minimal residual slough (Figures 2A-2B).

The patient was discharged on tapering doses of oral corticosteroids and advised regular follow-up with both rheumatology and ENT services. At subsequent follow-up visits, she remained symptomatically stable without recurrence of laryngeal symptoms.

## Discussion

SLE is a complex, multisystem autoimmune disease with diverse clinical manifestations [8]. Although mucocutaneous, musculoskeletal, renal, and hematologic involvement are well recognized, involvement of the larynx remains uncommon and frequently underdiagnosed [9]. The reported prevalence of laryngeal manifestations in SLE varies widely, ranging from subclinical mucosal inflammation to severe airway compromise. Because symptoms are often nonspecific, laryngeal involvement may be overlooked unless clinicians maintain a high index of suspicion, particularly when ENT symptoms occur in the context of systemic abnormalities.

Laryngeal involvement in SLE may arise through several pathogenic mechanisms. Immune complex deposition within mucosal tissues, small-vessel vasculitis, inflammatory edema, and arthritis of the cricoarytenoid joints have all been proposed as contributing factors [6]. These processes can result in a spectrum of structural changes, including mucosal ulceration, vocal cord edema, inflammatory masses, and hemorrhagic lesions. In some patients, inflammation of the cricoarytenoid joint may produce symptoms resembling vocal cord paralysis, while others may develop severe supraglottic edema leading to airway obstruction.

The wide variability in pathological mechanisms partly explains the heterogeneity in clinical presentation reported in the literature. Patients with laryngeal SLE most commonly present with hoarseness, dysphagia, odynophagia, cough, or throat discomfort [6]. In rare cases, stridor and acute airway compromise may occur, representing a medical emergency requiring prompt intervention. In the present case, the patient presented with acute-onset odynophagia and a change in voice, symptoms that are frequently attributed to more common inflammatory conditions of the upper airway. However, the absence of fever, respiratory infection, or local traumatic factors prompted further evaluation. Endoscopic examination plays a crucial role in identifying laryngeal involvement [10].

In this case, Hopkins rod endoscopy demonstrated significant supraglottic edema involving the epiglottis and aryepiglottic fold, along with a hemorrhagic bulla over the arytenoid and an ulcerative lesion extending along the lateral pharyngeal wall. Therefore, direct visualization of the larynx not only aids diagnosis but also helps assess the degree of airway compromise. An important feature in this case was the presence of severe hematological abnormalities, including leukopenia and profound anemia, which raised suspicion for an underlying systemic disorder. Hematologic manifestations such as leukopenia, anemia, and thrombocytopenia are well-recognized components of SLE and often serve as important diagnostic clues [11]. The presence of hepatosplenomegaly further suggested systemic involvement, prompting autoimmune evaluation. The strong positivity of antinuclear antibodies confirmed the diagnosis and allowed correlation of the laryngeal findings with underlying SLE. Another critical aspect of this case was the need to exclude infectious etiologies. Laryngeal lesions with ulceration and slough may resemble conditions such as tuberculosis, fungal infections, or malignancy, particularly in regions where infectious diseases remain prevalent. Careful clinical assessment and appropriate laboratory investigations are therefore essential to avoid misdiagnosis and inappropriate treatment. Management of laryngeal involvement in SLE primarily focuses on controlling systemic inflammation and preventing airway compromise.

Corticosteroids remain the cornerstone of therapy and typically lead to rapid clinical improvement. In the present case, systemic corticosteroid therapy combined with supportive care resulted in significant resolution of laryngeal edema and healing of mucosal lesions within a few days. The favorable response to steroid therapy further supports the inflammatory autoimmune nature of the laryngeal pathology. Early recognition and multidisciplinary management are essential to prevent potentially life-threatening complications. Otolaryngologists play a critical role in detecting early laryngeal manifestations, while collaboration with rheumatologists ensures appropriate long-term disease management. Timely intervention may prevent progression to airway obstruction and improve overall patient outcomes.

This case highlights the importance of considering systemic autoimmune diseases in the differential diagnosis of atypical laryngeal lesions. Awareness of such rare manifestations can facilitate early diagnosis and prompt treatment, thereby reducing morbidity. Although laryngeal involvement in SLE is uncommon, its potential severity underscores the need for heightened clinical vigilance.

## Conclusions

Laryngeal involvement in SLE, though uncommon, should be considered in patients presenting with unexplained laryngeal edema, ulceration, or voice changes, especially in the presence of hematological abnormalities. Early diagnosis and prompt initiation of immunosuppressive therapy are crucial to prevent airway compromise and ensure favourable outcomes. Otolaryngologists play a key role in the early detection of this rare but potentially life-threatening manifestation.
